# A case of a transient phrenic nerve paralysis after resection of a giant lymphangioma evaluated by dynamic digital radiography

**DOI:** 10.1186/s44215-023-00044-3

**Published:** 2023-06-14

**Authors:** Go Kamimura, Kazuhiro Ueda, Aya Takeda, Ryo Miyata, Masaya Aoki, Toshiyuki Nagata, Masami Sato

**Affiliations:** Department of General Thoracic Surgery, Graduate School of medical and Dental Sciences Kagoshima University, 8-35-1, Sakuragaoka, Kagoshima City, Kagoshima Pref 892-8520 Japan

**Keywords:** Dynamic digital radiography, Lymphangioma, Phrenic nerve

## Abstract

**Background:**

Lymphangioma is a relatively rare benign congenital malformation composed of dilated cystic lymphatic vessels; however, its occurrence in adults and manifestation as mediastinal lymphangioma are even rarer. In general, the incidence of mediastinal lymphangiomas has been reported to range from 0.01 to 4.5% of all mediastinal tumors. Mediastinal lymphangiomas tend to increase in size and can sometimes stretch the phrenic nerve that are present in the mediastinum, resulting in phrenic nerve palsy.

Dynamic digital radiography (DDR) allows for quantitative evaluation over time and has recently been utilized in actual clinical practice. The distance between the apex of the lung and diaphragm can be measured, and the movement of the left and right lungs and diaphragm can be evaluated separately. In this report, we describe a case in which DDR demonstrated the improvement of phrenic nerve palsy.

**Case presentation:**

A 32-year-old woman with no medical history and no smoking history reported dyspnea on exertion, and abnormalities were noted on chest X-ray. Close examinations revealed thymic, pericardial, bronchogenic, and teratoid cysts, and since they appeared to be growing in size, the patient was referred for surgery. Because the giant cyst prevented visualization of the thoracic cavity, puncture was performed for aspiration. The stretched phrenic nerve was identified in the giant cyst wall and was dissected to avoid damaging it. The cyst wall was detachable and excised without obvious mediastinal invasion. The patient had a good postoperative course and was discharged on postoperative day 5. Dynamic digital radiography on postoperative days 3 and 17 showed movement of the left diaphragm, with improvement over time.

**Conclusions:**

Mediastinal lymphangiomas tend to grow in size and can sometimes stretch the phrenic nerves that are present in the mediastinum, resulting in phrenic nerve palsy. Dynamic digital radiography (DDR) is a modality that can also be evaluated quantitatively and over time and can comprehensively determine improvement in transverse paralysis.

## Background

Dynamic digital radiography (DDR) allows for quantitative evaluation over time and has recently been utilized in actual clinical practice [1]. The distance between the apex of the lung and diaphragm can be measured, and the movement of the left and right lungs and diaphragm can be evaluated separately. We herein report a case of transient phrenic nerve paralysis after resection of a giant lymphangioma, which was evaluated by DDR.

## Case presentation

**Fig. 1:**
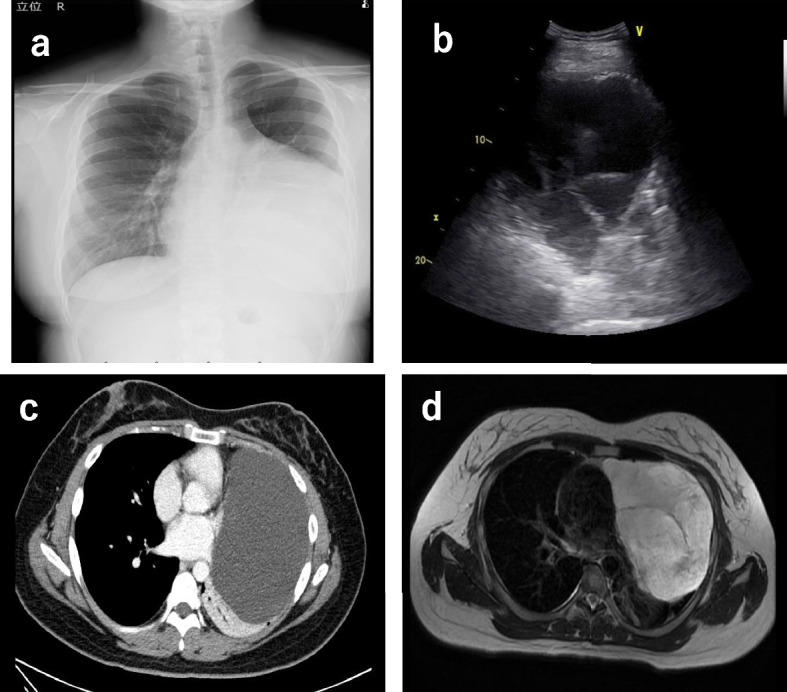
Preoperative imaging examination. **a** Chest X-ray revealed a huge tumor in the left lung field. **b** Body surface echocardiography revealed a multifocal cystic tumor. **c** Contrast-enhanced CT on a close examination indicated a massive cystic lesion 20 cm in size occupying the left thoracic cavity, contiguous with the mediastinum. **d** Contrast-enhanced MRI revealed a multifocal cystic tumor with no evidence of invasion into surrounding tissues

A 32-year-old woman with no medical history and no smoking history reported dyspnea on exertion, and abnormalities were noted on chest X-ray (Fig. 1a). Body surface echocardiography revealed a multifocal cystic tumor (Fig. 1b). A close examination with contrast-enhanced computed tomography (CT) showed a massive (20 cm) cystic lesion occupying the left thoracic cavity contiguous with the mediastinum (Fig. 1c). Contrast-enhanced magnetic resonance imaging revealed a multifocal cystic tumor with no evidence of invasion into surrounding tissues (Fig. 1d). ^18^F-fluorodeoxyglucose positron emission tomography showed no uptake in the tumor. These close examinations revealed thymic, pericardial, bronchogenic, and teratoid cysts, and since they appeared to increase in size, the patient was referred for surgery.

**Fig. 2:**
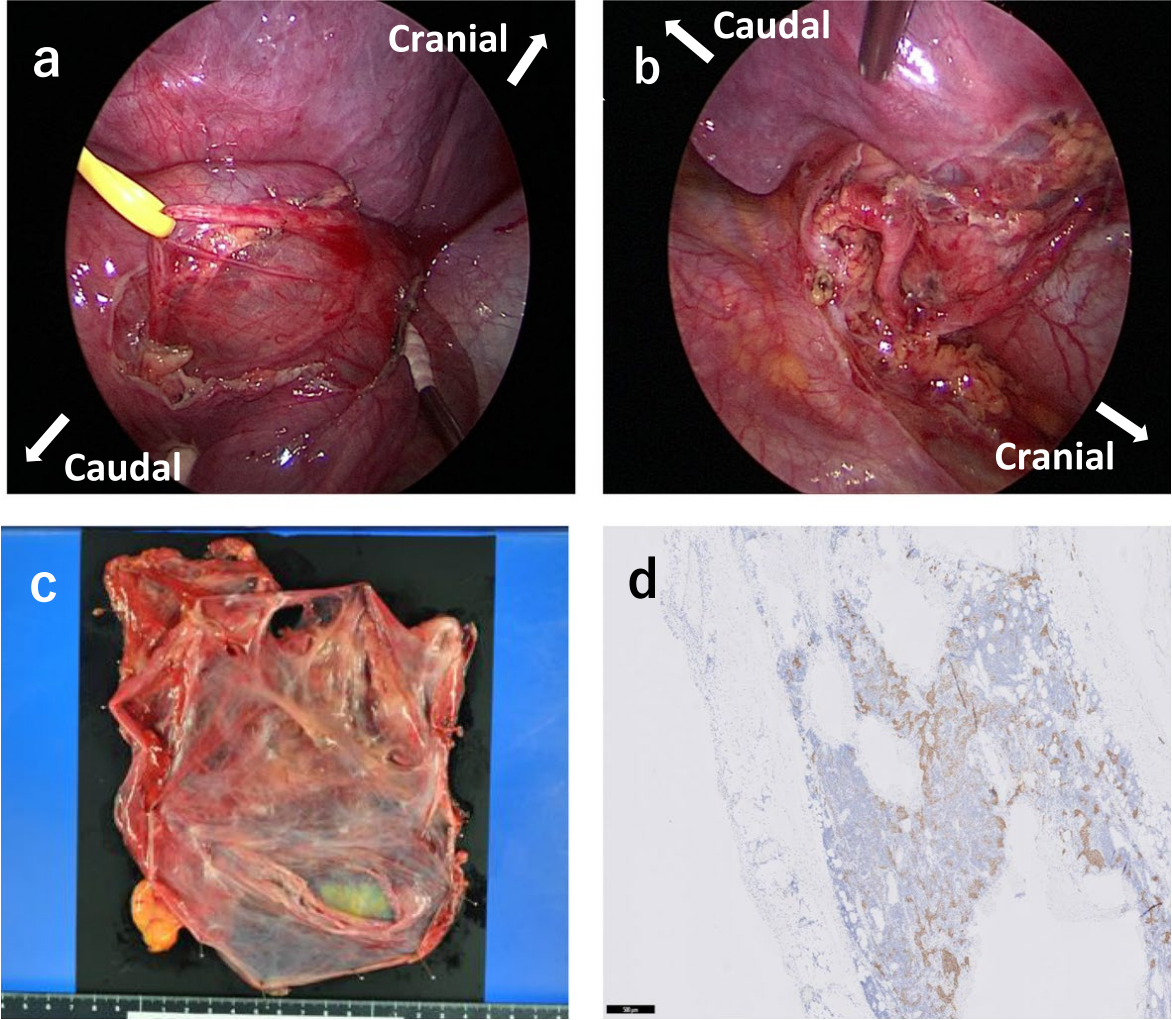
Surgical findings and pathology. **a**, **b** The stretched phrenic nerve was identified in the giant cyst wall and dissected so as not to damage it. **c** The cyst wall was detachable and excised without obvious mediastinal invasion. **d** The endothelial cells were immunostained positive for D2-40, a marker of lymphatic endothelium, and were diagnosed as cystic lymphangioma

The patient was placed in the right lateral decubitus position, and surgery was performed with four ports via a video-assisted thoracoscopic approach. Because the giant cyst prevented visualization of the thoracic cavity, puncture was performed for aspiration. The contents showed a pale-yellow serous liquid, an analysis of which revealed the following composition: LD (International Federation of Clinical Chemistry and Laboratory Medicine [IFCC]), 160 U/l; Na, 140 mEq/l; Cl, 109 mEq/l; K, 3.6 mEq/l; and TP, 4.7 g/dl. This was almost the same composition as the plasma components. The volume of the internal solution was 1850 ml. The stretched phrenic nerve was identified in the giant cyst wall and dissected to avoid damaging it (Fig. 2a, b). During debridement, great care was taken to avoid causing thermal damage via electrocautery. The cyst wall was detachable and excised without obvious mediastinal invasion (Fig. 2c). A pathological examination revealed that the cyst was covered with a layer of flattened endothelial cells, and the wall was composed of sparse connective tissue and smooth-muscle fibers. Immunostaining of the endothelial cells was positive for D2-40, a marker of lymphatic endothelium. Based on these results, a diagnosis of cystic lymphangioma was made (Fig. 2d).

**Fig. 3:**
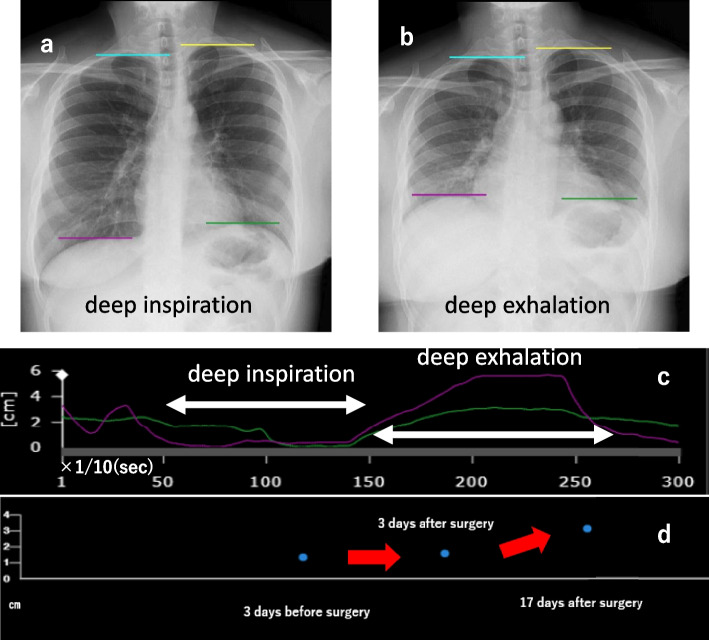
Details of dynamic digital radiography image. **a** Dynamic digital radiography image during deep inspiration 17 days after surgery. **b** Dynamic digital radiography image during deep exhalation 17 days after surgery. **c** Displacement of the left and right diaphragm over time during a single breath. **d** The patient showed improvement in left diaphragmatic movement over time

The patient had a good postoperative course and was discharged on postoperative day 5. Dynamic digital radiographs on the third postoperative day after the drain was removed and on day 17 of the post-discharge outpatient visit showed movement of the left diaphragm, which improved over time (Fig. 3a–d). From the postoperative period, her respiratory distress gradually improved. On preoperative respiratory function tests, the vital capacity (VC) was 2.49 L and the forced expiratory volume 1.0 (FEV1.0) was 1.96 L. On the 17th postoperative day, the VC was 2.98 L and the FEV1.0 was 2.40 L, indicating improvement.

## Discussion

Lymphangioma was first described by Redenbacker in 1828 as a disease of unknown cause [2]. The occurrence of lymphangioma in adults and manifestation as mediastinal lymphangioma are rarer. In general, the incidence of mediastinal lymphangiomas has been reported to range from 0.01 to 4.5% of all mediastinal tumors [3, 4]. Mediastinal lymphangiomas tend to grow in size and can sometimes stretch the phrenic nerves that are present in the mediastinum, resulting in phrenic nerve palsy. Tumors tend to grow and bleed within the cyst, and there have been reports of malignant transformation; thus, complete resection is considered the basic treatment [5, 6].

Reports on the removal of lymphangioma as massive as that in the present case are rare. Because the tumor is large enough to occupy the thoracic cavity, the surgical field is secured by suction to prevent leakage of the internal solution. The vulnerable phrenic nerve, which is stretched by the tumor, must then be dissected without damaging it.

The phrenic nerve is an important nerve for the respiratory function, and phrenic nerve injury can cause breathing problems. In some cases, phrenic nerve surgery may be required to restore the diaphragmatic function [7].

DDR acquires images using a flat panel detector that supports moving images and an X-ray tube capable of continuous pulse irradiation. This technique allows for the continuous acquisition of chest X-ray with high temporal resolution during breathing [1]. It is also effective in actual clinical practice and has been rapidly implemented in recent years. In addition, CT is performed in the supine or prone position, while dynamic chest radiography can be performed in the standing or sitting position, reflecting physiologically relevant daily activities. The amount of DDR radiation dosage is double that of chest X-ray, one-twentieth that of CT (perfusion scan of DDR, 0.2 mSv vs. chest X-ray, 0.1 mSv vs. CT, 10 mSv) [8].

Various analyses can be performed with DDR. Post-processing using a software program allows visualization of the temporal changes in radiograph translucency, which represent changes in the pulmonary circulation due to cardiac pumping. DDR can diagnose acute pulmonary thromboembolism without the use of computerized tomography or contrast media [9]. It is also possible to determine the parts of the lung fields that are more ventilated by extracting the concentration changes in the lung fields associated with breathing.

DDR also allows for quantitative evaluations over time. Diaphragm motion tracking processing can discriminate the apex of the lungs from the apex to the lung bases and automatically recognizes the degree of diaphragm movement individually, quantifying it in the vertical direction (Fig. 3a–d) [1]. Typically, we evaluate phrenic nerve palsy by comparing chest X-rays taken at deep inspiration and deep expiration. The phrenic nerve palsy is evaluated by comparing only the height of the diaphragm at deep inspiration and deep expiration.

However, DDR is very beneficial because it allows for real-time observation of diaphragm movements, including strange movements during breathing that cannot be seen on chest X-ray, and even the coordinated movements of the diaphragm on both sides. As in the present case, the bilateral diaphragm may show independent respiratory motion between inhalation and exhalation without synchronized respiratory motion between the diaphragm on each side (Fig. 3). Such diaphragmatic respiratory motion, which can never be assessed by chest X-ray alone, may lead to a sense of dyspnea.

Marino et al. [10] reported that pulmonary hyperinflation due to the exacerbation of chronic obstructive pulmonary disease causes diaphragmatic descent and flattening, which physically damages the axons of the phrenic nerve. The electrical conductivity defect then causes diaphragmatic dysfunction. However, they report that the damage is usually repairable and that the phrenic nerve damage gradually improves as the disease improves.

In this case, DDR was utilized to confirm the function of the stretched phrenic nerve caused by the giant cyst. On postoperative day 3, tumor resection showed improvement of the chest pressure sensation; however, her symptoms of respiratory distress remained. Subsequently, the patient was reevaluated by DDR on day 17 and showed improvement in diaphragmatic movement over time as well as improvement in dyspnea.

The stretched phrenic nerve in our patient was preserved, and the giant lymphangioma was removed. DDR confirmed that the phrenic nerve function had improved over time. DDR is useful for real-time and quantitative assessment of diaphragmatic respiratory motion.

In the future, as more cases are accumulated, it will be useful to not only compare the diaphragm height, but also to focus on the respiratory motion of the diaphragm. This will clarify the relationship between respiratory function test results and DDR, and the characteristics of DDR that cannot be evaluated by chest X-ray.

## Conclusion

We experienced a case of tumor resection while preserving the phrenic nerve that had been stretched by a giant lymphangioma. We then evaluated the function of the preserved phrenic nerve by DDR. DDR is useful for real-time and quantitative assessment of diaphragmatic respiratory motion.

## Data Availability

Data that support the findings of this study are not openly available due to [reasons of sensitivity, e.g., human data] and are available from the corresponding author upon reasonable request [include information on the data’s location, e.g., in a controlled access repository where relevant].
